# Use of Socially Assistive Robots in Physiotherapy: Scoping Review

**DOI:** 10.2196/69908

**Published:** 2025-09-03

**Authors:** Jiaxin He, Melanie K Farlie, Pamela Carreno-Medrano

**Affiliations:** 1 Department of Physiotherapy, Faculty of Medicine, Nursing and Health Science, Monash University, Melbourne, Australia; 2 Monash Centre for Scholarship in Health Education, Faculty of Medicine, Nursing and Health Science, Monash University, Melbourne, Australia; 3 Department of Electrical and Computer Systems Engineering, Faculty of Engineering, Monash University, 18 Alliance Lane, Melbourne, 3800, Australia, 61 399055562

**Keywords:** socially assistive robots, robotics, physical therapy modalities, rehabilitation, human-robot interaction

## Abstract

**Background:**

Socially assistive robots (SARs) are robotic technology platforms equipped with sensing (eg, through audio or visual) and acting (eg, speech and movement) capabilities to interact socially with users. SARs are increasingly adopted in physiotherapy to aid patients in their rehabilitation journey by providing feedback, motivation, and encouragement. However, while many studies have explored SAR implementation in physiotherapy, research involving clinical populations remains scarce, and the overall state of SAR deployment is unclear.

**Objective:**

This scoping review aimed to explore the use of SARs in physiotherapy with clinical populations, how the effectiveness of these interventions has been evaluated, and identify limitations and new areas of application and future work.

**Methods:**

Following the PRISMA-ScR (Preferred Reporting Items for Systematic Reviews and Meta-analyses for Scoping Review) reporting guideline extension, comprehensive searches based on SARs and physiotherapy were conducted in various databases. Title and abstract screening were performed by 1 reviewer, with full-text screening conducted by 2 reviewers. Data extraction, synthesis, and analysis were completed by 1 reviewer. Data on SAR roles were categorized and synthesized using content analysis. Other descriptive texts were summarized to improve readability.

**Results:**

Our findings suggest that SARs are commonly used in rehabilitation clinics and hospital inpatient settings, primarily for neurological conditions. In these interventions, SARs typically serve roles, such as coaching, demonstration, monitoring, and peer support. Their effectiveness is generally evaluated through clinical outcomes, user performance, functional measures, and metrics assessing the robots’ acceptability, usability, and perception.

**Conclusions:**

This scoping review highlighted SARs’ potential to address challenges faced by human therapists due to the demands of time-extending coaching and monitoring and the limited availability of therapists. Future research should focus on addressing the limitations identified in this scoping review, including small sample sizes, technical issues in both the robot and intervention design, sufficient involvement of key stakeholders in the design and development of SAR-based interventions, and conducting more clinical trials to investigate SAR intervention effectiveness.

## Introduction

Socially assistive robots (SARs) are designed to provide support to users primarily through social interaction and engagement. SARs were first defined and introduced in the field of physiotherapy in a foundational publication by Feil-Seifer and Mataric [1]. Unlike physically assistive robotic systems, SARs can achieve their primary function without performing any physical work or physically interacting with the environment or the intended user [2]. SARs are endowed with unique capabilities in human-social interactions, such as recognizing patterns of human interactions and engaging users through various forms of communication, including verbal and nonverbal cues, such as motion, and visual or tactile aids [1,3]. By emphasizing noncontact social interaction, SARs have shown promise in enhancing patient adherence, motivation, and engagement in rehabilitation exercises [4]. These merits in SAR application are the key to assisting physiotherapists in fostering active participation in health care programs [5].

In rehabilitation, where time spent engaging in interventions is a key dosage parameter, SARs play an important role by offering verbal encouragement, coaching, feedback, reminders, and continuous monitoring to enhance the overall therapeutic intervention or self-directed therapy [5,6]. For instance, for children diagnosed with cerebral palsy (CP), conventional therapy approaches may benefit from novel strategies to increase motivation and thus achieve optimal outcomes [7]. A previous review has found that children and young adolescents with CP generally interact positively with SARs, leading to improved motivation and engagement [8]. Furthermore, SARs can be tailored to conduct gamified motor training sessions, capturing the attention of children with CP and encouraging active participation [9]. SARs’ ability to address barriers to participation in therapy, such as motivation and self-efficacy, aligns with goals recognized by the International Classification of Functioning, Disabilities and Health (ICF) [10] developed by the World Health Organization (WHO), which serves as the most encompassing taxonomic model to describe an individual’s functioning and disability [11].

While existing literature indicates that SARs show promise for improving outcomes across health and social care settings for diverse populations, their specific applications in physiotherapy remain poorly characterized [12]. Despite the growing interest in integrating SARs into rehabilitation settings, the full breadth of usage in physiotherapy practice remains unclear, partly due to a current lack of literature summarizing SAR-assisted physiotherapy studies directly involving clinical populations. Existing reviews of SAR-assisted therapy in physiotherapy have primarily focused on interventions for children living with CP, providing valuable yet constrained perspectives on the potentially broader usage of SARs in physiotherapy practice [9,13,14]. A scoping review exploring the use of robot-assisted therapy for upper limb impairments in children with CP identified 1 type of SAR (CosmoBot) [14]. This SAR was used in a small sample size (n=6) study investigating effects on functional movement quality, thus providing a limited view of the state of SAR deployment in physiotherapy practice.

This paper aims to fill this gap and advance the field by presenting a scoping review of the current state of the art of SAR deployment in physiotherapy with clinical populations [15]. Specifically, this scoping review seeks to identify how SAR systems are implemented and operationally integrated into interventions and activities reported in physiotherapy contexts and describe the methods used to evaluate the effectiveness of interventions that integrate the use of SARs. In addition, the review aims to identify current limitations to SAR integration within physiotherapy interventions and explore new potential application areas suggested in research to date. The findings of the scoping review will guide future interdisciplinary collaboration between physiotherapy, roboticists, and human-robot interaction researchers interested in investigating SAR applications in clinical populations.

## Methods

### Study Design

This review was a systematic scoping review that followed the methodological steps first described by Arskey and O’Malley [16] and subsequently refined by Levac et al [17], Peters et al [18] and Pollock et al [19]. A scoping review was determined to be the most appropriate review methodology given the paucity of clinical trials investigating the effectiveness of SAR interventions in clinical physiotherapy, which precludes the conduct of a systematic review [20]. As such, our review question was “What is known about the use of SARs within physiotherapy interventions and activities with clinical populations?”

The advantages of a scoping review approach are the development of a systematic and reproducible search strategy, with clear inclusion and exclusion criteria to comprehensively identify and chart existing evidence related to our review question and identify gaps and potential avenues for future research. This scoping review report follows the PRISMA-ScR (Preferred Reporting Items for Systematic Reviews and Meta-analyses for Scoping Review) [21]. Given the nature of the scoping review being a type of systematic review, no ethical approval was needed.

### Search Strategy

The scoping review search terms were structured using the population, concept, context (PCC) framework [18]. For this review, the population was any clinical population, the concept of interest was SARs, and the context of interest was the conduct of physiotherapy interventions or activities. To ensure a balanced and comprehensive yield of existing publications related to robotics in physiotherapy, a combination of health and engineering literature databases was systematically searched. The engineering databases searched for this scoping review were the Association of Computing Machinery (ACM) and the Institute of Electrical and Electronics Engineers (IEEE) databases. The health databases searched were Ovid MEDLINE, Ovid Emcare, Ovid EMBASE, Ovid Cochrane Library, Scopus, and the CINAHL.

A primary database search string was developed in consultation with an academic librarian as recommended for scoping reviews [18]. The first search was constructed for execution in MEDLINE using Medical Subject Headings (MeSH), database-specific thesaurus equivalent words and keywords for SAR (eg, “social* assist* robot”) and physiotherapy (eg, “physiotherapy”). Details of complete syntax are listed in (Multimedia Appendix 1).

The primary search string was then adapted for use in all other electronic databases by modifying syntax and using database-specific subject headings (ie, MeSH). In cases where a database did not use subject headings (such as Scopus, ACM, and IEEE), only keywords were used. A study date limit filter was applied to restrict the search for publications since the foundational 2005 publication of Feil-Seifer and Mataric [1]. All databases were searched up to November 23, 2023. Complete search strings for all databases searched are presented in (Multimedia Appendix 2).

Identified records from all databases were uploaded to reference manager software Endnote (Clarivate; [22]) and imported into systematic review software Covidence (Veritas Health Innovation; [23]) for screening. Records were automatically deduplicated in Covidence before conducting title, abstract, and full-text screening. Further manual identification and removal of duplicates were conducted during the screening stages to ensure no duplicate records appeared in the final yield.

### Inclusion and Exclusion Criteria

#### Population

All studies involving clinical populations interacting with SARs were included.

#### Concept

Only studies reporting on SARs as an intervention, a medium to deliver an intervention, or both were considered. As defined in the introduction, the SARs reported must be capable of providing noncontact social interaction with users. Therefore, studies involving surgical or physically assistive robotics, artificial intelligence software, and virtual intervention without focusing on SARs were excluded.

#### Context

Studies conducted using physiotherapy as an intervention or cotherapy in various settings, such as hospitals, clinics, community health care centers, and residential aged care facilities, were included.

#### Types of Studies

Only full-text peer-reviewed primary studies written in English and published from 2005 to the present were included. Peer-reviewed conference papers and proceedings reporting completed studies were considered for inclusion, as in the engineering literature, these undergo a peer review process akin to that of journal papers in the medical field. Any unfinished studies and gray literature, including short technical reports and workshop papers, were excluded. Only studies that reported clinical studies were considered. Publications that solely described robot design and development or evaluated system feasibility, system features, or user perception were excluded.

### Selection of Relevant Publications

All references were imported into Covidence [23]. One reviewer (JH) screened all records at the title and abstract stage against the eligibility. To confirm reviewer accuracy in applying the eligibility criteria, a subset of 25 studies was randomly selected for independent evaluation by all 3 review team members. The outcome of the 3 independent reviews of the 25 studies was compared and agreement on the screening outcome was confirmed.

Two reviewers independently screened all papers at the full-text screening stage. One reviewer (JH) screened all papers while the second independent review was conducted by either MF or PCM. Meetings between all 3 reviewers occurred at regular intervals during all screening stages, ensuring close supervision of JH’s screening progress and a unified and consistent interpretation of the criteria. Conflicts identified at the full-text screening stage were resolved by an initial discussion between the reviewing pair (JH and MF or JH and PCM) until a consensus was reached. If a consensus could not be reached by the reviewing pair, the third reviewer was consulted and discussion continued until a consensus was reached. Included studies advanced to data extraction, while exclusion reasons were documented for excluded studies.

### Data Extraction

A data-charting form was developed in Covidence [23] to extract relevant data from the included studies [4,24-35] . The types of data extracted are shown in (Table 1). The review team piloted the data-charting form with the first 2 records and revised it before charting the data from the rest of the included studies [4,24-35].

One of the reviewers (JH) completed the remaining data extraction process to ensure data extraction consistency. Two reviewers (MF and PCM) audited the extracted data from 4 included studies [26,30,34,35] (approximately 30% of the final yield) and identified negligible errors in the data extracted. Extracted data were then exported from Covidence [23] to a spreadsheet for synthesis.

**Table 1.** Template for extracting information from the included studies.

Topic	Extraction details
Study	Title, author name, publication year, country, study duration, study design, study aim, and sample size.
Population	Age and condition or diagnosis.
Concept	Physiotherapy setting, physiotherapy goal, type of training, specific exercise prescribed to patients, length of each session in minutes, number of sessions, and intensity of training.
Context	Name of the robot, level of autonomy, if the robot was being controlled during the intervention, technical support presence during the intervention, area of physiotherapist and patient or client involvement in the protocol or robot design phase, robot model, the function of robot, the behavior of the robot, the communication channels between robot and users, presence of physiotherapist during the intervention, role of physiotherapist and presence of a control group.
Outcome and finding	Construct investigated and associated outcome measure, key finding, study and robotic limitation, and area of application.

### Data Analysis and Synthesis

Data synthesis and analysis was led by JH with regular review meetings with MF and PCM to confirm the accuracy of the synthesis process and devise a shared interpretation of the data synthesized. Charted data were first categorized into groups related to the scoping review questions: study and participant characteristics (population), features of physiotherapy (context), features of SARs (concept) used in interventions, robot design features, evaluation methods, key findings, limitations, and areas of application. Patterns were identified within each group and categorized, which aided the comparison and interpretation of data.

Subsequently, a more detailed analysis of the data extracted from included studies [4,24-35] by applying content analysis [36] was performed. Descriptive text extracted from studies relating to robot function and behavior was condensed, and patterns related to robot functions were coded and grouped into categories. Similarly, descriptive text concerning the role of physiotherapists, physiotherapists, and patient or client involvement in the protocol and robot design phase, key findings, limitations, and application areas were condensed, coded, and categorized. Throughout the data analysis process, all data were directly linked to the source material, and where necessary, the original text was revisited to confirm context or meaning if extracted data segments were ambiguous when being analyzed in isolation.

## Results

### Description of Included Studies

**Figure 1.**
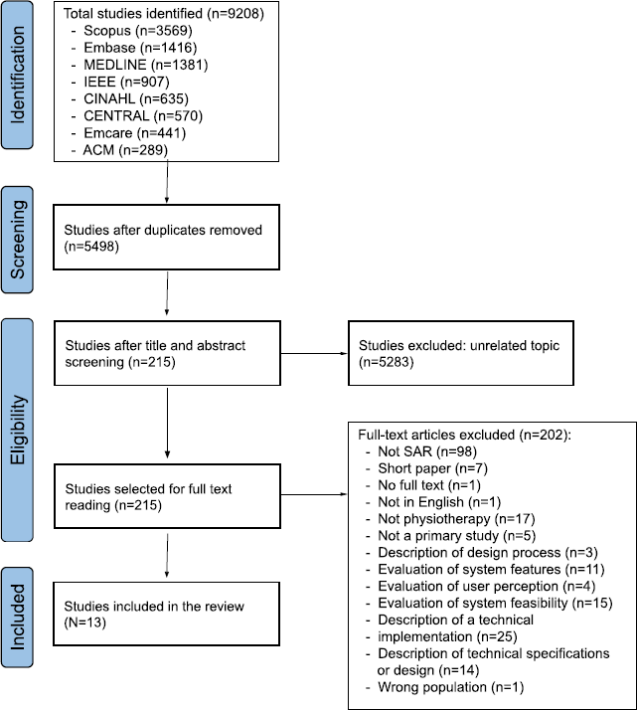
Flowchart of study selection process adapted from the PRISMA (Preferred Reporting Items for Systematic Reviews and Meta-analyses for Scoping Review) flowchart for this review.

The database searches identified 9208 studies. After removing duplicates (3710 studies) and title and abstract screening, 215 studies were included for full-text screening. Of those, 202 studies were excluded with main reasons shown in Figure 1. Seven short papers related to our review question were identified at the full-text screening stage. A citation linkage search using Google Scholar was conducted to establish if any subsequent full-text reports had been published. No additional records meeting the inclusion criteria were identified by citation tracking of the studies included at the full-text stage. A final yield of 13 studies [4,24-35] met the criteria for inclusion in the review (Figure 1).

### SAR-Related Interventions and Activities in Physiotherapy

In this section, we begin by summarizing the general characteristics and demographic details of the selected studies. Following that, we provide an overview of the specific physiotherapy interventions, including prescribed exercises, dosage, and objectives.

#### Physiotherapy Settings and Participant Groups

Table 2 lists the main characteristics, demographic details, and countries where the SAR interventions took place. For studies that did not explicitly report their country of implementation, we derived the location from the institutional affiliation of the first or corresponding author, treating this as the proxy country of study. Among the included studies, the geographical distribution was as follows: Colombia hosted the largest number (3/13, 23%) [24-26], followed by 2 studies each in Spain [27,28], Israel [29,30], and Australia [4,31]. Single studies were reported from Belgium [32], France [33], and Saudi Arabia [34].

**Table 2.** Description of included studies with information is categorized by physiotherapy settings where socially assistive robotics were used.

Article and Year	Country	Study duration	Study design	Sample size	Study group (s) and age in years (mean, SD)	Diagnosis or Condition
Hospital inpatient						
Pulido et al [27], 2019	Spain	4 months	Quasi-experimental small-N design	8	Traditional group: 3-10 Robot group: 3-10	Obstetric brachial plexus palsy or infantile cerebral palsy
Salas-Monedero et al [28], 2023	Spain	Not reported	Prospective observational study	10	Patients with tetraplegia: 9.7 (4.0) Patients with paraplegia: 10.6 (2.1)	Pediatric-onset chronic spinal cord injury
Meyns et al [32], 2019	Belgium	8 months	Cohort study	89 (typical developing children: 75; children with oncological disorders: 14)	Typical developing children: 4-13 (mean 7.9, SD 2.3), Children with oncological disorders: 3-15 (mean 7.0, SD 3.8)	Neutropenia and cancer
Both inpatient and outpatient						
Butchart et al [31], 2021	Australia	2 months	Not reported	10 (children: 5; parents: 5)	Children: 6-12 Parents: 38-47	Cerebral palsy, hemispherectomy, or hereditary spastic paresis
Rehabilitation center						
Buitrago et al [24], 2020	Colombia	8 weeks	Case report	1	8	Dyskinetic cerebral palsy
Feingold Polak and Tzedek [29], 2020	Israel	5-7 weeks	Not reported	4	51-75 (mean 64, SD 10)	Stroke
Cespedes et al [25], 2020	Colombia	Not reported	Not reported	4	Between 20 and 60	Stroke, spinal cord injuries, and Guillain-Barré syndrome
Blanchard et al [33], 2022	France	19 months	Randomized controlled trial	27 (robot: 15; control group: 12)	Robot group: 42.9 (12.8) Control group: 43.8 (9.7)	Nonspecific low back pain
Rehabilitation clinic						
Carrillo et al [4], 2020	Australia	23 months	Not reported	9	Not reported	Cerebral palsy
Feingold-Polak et al [30], 2020	Israel	5-7 weeks	Randomized controlled trial	24 (robot group: 11; computer group: 13)	Robot group: 30-76 (mean 54.8, SD 13), Computer group: 40-77 (mean 62.6, SD 15)	Stroke
Irfan et al [26], 2020	Colombia	18 weeks	Longitudinal case study	1	6	Myocardial infarction
Al‑Shahry and Al‑Shehri [34], 2020	Saudi Arabia	5 trials on different days	Not reported	5	20-65	Patients with musculoskeletal conditions diagnosed with pain in muscles or joints or both
Private nursing home						
Bäck et al [35], 2013	Finland	7 months	Not reported	34	Median age greater than 85	Residents of the nursing home

SAR interventions were implemented across various physiotherapy settings, with the most common being rehabilitation clinics [4,24-26,29,33-35] (8/13, 62%) and hospital inpatient settings [27,28,32] (3/13, 23%). One study [31] reported rehabilitation in both inpatient and outpatient settings (1/13, 8%). Inpatient physiotherapy takes place when the patient is admitted to the hospital, while outpatient physiotherapy occurs outside of hospital admission, typically in outpatient departments of hospitals, rehabilitation centers, or private clinics. In addition, 1 study reported SAR interventions in a private nursing home [35].

Regarding the context of application, we found that the reported SAR interventions were predominantly applied in the treatment of neurological conditions, such as CP, obstetric brachial plexus palsy, spinal cord injury, hemispherectomy, hereditary spastic paresis, stroke, and Guillain-Barre syndrome [4,24,25,27,29-32] (8/13, 62%). Musculoskeletal conditions (eg, low back pain, muscle or joint pain) were next in frequency [33,34] (2/13, 16%), followed by cancer [32] (1/13, 8%) and cardiovascular conditions (eg, myocardial infarction) [26] (1/13, 8%). One study’s participants were residents in an older care facility [35].

The age range of SAR intervention recipients varied from 3 years old to more than 85 years. Specifically, 4 studies [24,27,28,32] targeted children (younger than 18 years of age), including those in early childhood and adolescence. Notably, 7 studies [25,26,29,30,33-35] focused on adults (18 years and older), encompassing a broad range of ages within this group. Overall, 1 study [31] included both children and parents in the intervention. In addition, 1 study [4] did not report the age of the intervention recipients.

Out of 13 included studies [4,24-35], only 5 studies [27,28,30,32,33] reported using a control group. This distinction is important, as control groups are critical to determining whether the observed effects are truly due to the inclusion of SARs.

#### Features of Physiotherapy Intervention

Table 3 lists all the physiotherapy interventions extracted from the studies. Interventions encompassed various types of training, specific exercises or programs, frequency, timings, and intensities of training, as well as physiotherapy goals tailored to specific patient populations.

**Table 3.** Description of physiotherapy intervention with information is categorized by the type of physiotherapy training.

Author and year	Specific exercises	Physiotherapy goals	Frequency and time	Intensity
Endurance training				
Irfan et al [26], 2020	Warm-up (stretching), exercises on a treadmill, and cool-down (low-intensity exercises)	Accelerate recovery and reduce the risk of recurrent events.	35×20‐30-minute sessions for 18 weeks.	Relative intensity of the sessions, determined by the treadmill speed and inclination, was progressively increased over time.
Salas-Monedero et al [28], 2023	Warm up, seated upper extremity muscle exercises (shoulder abduction, shoulder flexion-extension, elbow flexion-extension, and elbow elevation), and cool down	Enhance strength and endurance requirements in preparation for repetitive movements, such as those used in neurorehabilitation to acquire movement patterns for activities of daily living.	30-minute sessions, 2-3 times per week for a maximum of 5 weeks (10 sessions in total).	Not reported
Carrillo et al [4], 2018	Sit-to-stand exercise, exercise from a lying down position, and toy relay game	Delivery and participation in exercise programs in pediatric rehabilitation.	1 session of up to 30 minutes per participant.	Not reported
Strength training				
Bäck et al [35], 2013	Arm and leg exercise in sitting	Delivery and participation in a group exercise program.	4 sessions of 10-20 minutes each.	Not reported
Al‑Shahry and Al‑Shehri [34], 2020	Strengthening exercise	Improve back strength.	5 sessions of 30 minutes each.	Not reported
Butchart et al [31], 2021	Strengthening exercises in supine (such as bridges) and side-lying (such as hip abduction), relay games with obstacles	Delivery and participation of exercise programs in pediatric rehabilitation.	Up to 3 sessions, average session length of 33 minutes.	Not reported
Functional task training				
Feingold-Polak et al [30], 2021	Reach-grasp-and-place, or reach-grasp-and-manipulate movements, using real everyday objects, such as cups, jars, keys, wallets, and drawers.	Use functional task training to practice force regulation and retraining coordination.	30-50 minutes per session, 2-3 times per week for 5-7 weeks (15 sessions in total).	Not reported
Feingold Polak and Tzedek [29], 2020	Kitchen game (arrange jars on shelves at 3 different heights), cup game (ordering a set of colored cups in a row according), target game (ordering a set of colored cups in a circle), keys game (ordering a set of colored keys on a board), drawer game (take objects out of drawers located at different heights), card game (manipulation of cards), and wallet game (take the keys out of zipped wallets)	Retraining coordination of reach-to-grasp movements.	40-60 minutes per session, 2-3 times per week for 5-7 weeks (15 sessions in total).	Not reported
Gait training				
Cespedes et al [25], 2020	Walking using Lokomat	Promote functional locomotor recovery; retrain gait through neuroplasticity stimulation.	SAR^a^-assisted therapy: 1x45-minute session; conventional therapy: 1x 45-minute session	Not reported
Gross motor and motor control training				
Meyns et al [32], 2019	Gross motor movement exercises, such as raising arms, squats, lunges, and walking forwards and backwards	Participating in physical exercises.	1-minute set of exercise, 1-3 sets for each of the 4 conditions (each participant was asked to perform in all 4 conditions).	Not reported
Buitrago et al [24], 2020	Walking	Improve functional movement and cognitive skills.	16×45-minute sessions over 8 weeks.	Not reported
Flexibility and proprioception training				
Pulido et al [27], 2019	Mirror game (imitate different poses) and imitation game (imitate a sequence of poses in a specific order)	Training proprioception and improving joint mobility ranges	SAR-based therapy: 30 minute session, twice per week for 2 months, maximum of 15 sessions for each participant; traditional therapy: same as above	Not reported
Blanchard et al [33], 2022	Stretching	Promote participation and adherence to long-term exercise therapy for people with chronic low back pain	30-minute sessions, 5 times per week for 3 weeks (15 sessions)	Not reported

a SAR: socially assistive robotic.

SARs were used across a range of training modalities, including endurance training [4,26,28] (3/13, 23%), which focused on improving cardiovascular fitness; strength training [31,34,35] (3/13, 23%), aimed at increasing muscle strength; functional task training [29,30] (2/13, 15%), which helped patients practice real-life activities; gait training [25] (1/13, 8%), targeting walking ability and balance; gross motor and motor control [24,32] (2/13, 16%), which improved muscle movement and coordination; and flexibility and proprioception training [27,33] (2/13, 16%), aimed at enhancing joint range of motion and the body’s ability to sense movement and position.

The specific exercises described in the interventions varied based on the targeted condition and goals. Setting clear goals is crucial for guiding interventions toward desired outcomes and requires a thorough understanding of the patient’s activities, needs, limitations, and strengths [37]. The goals in SARs interventions can be grouped into 2 main categories: increasing activity and participation, as seen in studies by Carrillo et al [4], Back et al [35], Butchart et al [31], Meyns et al [32], and Blanchard et al [33]; and targeting underlying impairments in patients’ physical function, such as improving strength and endurance [28], enhancing back strength [34], and retraining coordination [29,30], gait [25], and proprioception [33].

In addition to the conventional therapeutic exercises (eg, treadmill exercise, robot-assisted gait training, and stretching) reported in the majority of the studies [4,24-26,28,32,34,35], SARs were also involved in gamification programs, as reported in 3 studies [27,29,30]. While all studies provided sufficient details on the exercise programs’ frequency and time parameters, only 1 study [26] specified exercise intensity. Reporting on these parameters, such as frequency, duration, and intensity is crucial, as it helps track progress and ensures that programs are tailored to the individual’s needs and capabilities.

Furthermore, 7 studies [24,26-30,33] implemented relatively long-term physiotherapy interventions, with session numbers ranging from 10 to 35 and total duration spanning from 3 to 18 weeks. In contrast, 3 studies [31,34,35] conducted 5 or fewer sessions, and 3 studies [4,25,32] administered only 1 therapy session per participant.

### Finding on SARs Characteristics

To answer the question of how SARs are integrated into physiotherapy contexts, we categorized the information on SARs characteristics based on 4 subquestions.

1. What SAR platforms are most used in physiotherapy?

2. How do SARs and users interact with each other?

3. What roles do these robots serve within physiotherapy contexts?

4. What was the involvement of key stakeholders in the design and development process of SARs and intervention protocols, as well as the presence of technical support and the role of physiotherapists during interventions?

This information aims to provide insights into the relationship we seek to build between robots and users, and how to ensure that SAR interventions are designed with the needs of target users in mind, thereby promoting broader community adoption.

#### Types and Levels of Autonomy of SARs in Physiotherapy

All reported SARs were humanoid robots, designed to mimic the human body in shape and capable of precise movements for tasks, such as demonstration. Among these, NAO was the most frequently used robot platform (10/13) [4,24-28,31,32,34,35]. NAO is a programmable humanoid robot developed by Aldebaran Robotics (SoftBank Robotics) [27]. Pepper, also developed by Aldebaran Robotics (SoftBank Robotics), was the second most commonly used robot [29,30]. One study [33] used Poppy, an open-source humanoid robot developed based on the Poppy project [38].

Using the classification by Beer et al [39], we identified 3 categories of robot autonomy from the included studies: “fully autonomous,” “semiautonomous,” and “tele-operated.” One study [26] reported that the SAR was “fully autonomous,” indicating that the robot operated independently without human intervention. In contrast, 2 studies [29,30] reported that the SAR was “semi-autonomous,” as the robot required some degree of human input or supervision during the intervention. However, the majority of studies [4,24,25,27,28,31-35] (10/13, 77%) did not provide a specific description of the level of autonomy or how the robot was operated.

#### Interaction Between SARs and Users

Eleven studies [4,24-27,29-31,33-35] reported several communication channels from robots to users during the physiotherapy interventions and activities. The use of speech or verbal feedback was the most commonly reported communication method [4,24-27,29,31,33-35] (11/13, 85%). In most studies, the robot used audible phrases with a synthetic voice or prerecorded instructions to engage patients, communicate with the physiotherapist, or provide verbal feedback on the timing or success of tasks [25,29,35]. Motion or gesture was also commonly used, reported in 10 studies [4,24-27,29,31,33-35] (10/13, 77%). For instance, the robot could bow to demonstrate correct spinal posture [25], imitate the patient’s movements to demonstrate exercises [27], or perform physical gestures, such as hand clapping or head nodding, to motivate patients [29]. In addition, visual feedback was provided by displaying messages or images on the robot’s tablet screen [30,33], and LED outputs were used to prompt inputs [4].

Users were able to command the robots through various methods, including touching the robot’s head-based tactile sensors [4,24,26], using voice commands to stop, resume, and restart the exercise program [34], pressing a physical button on a table to indicate completion of exercises [29,30], and interacting with a tablet interface [26]. However, 7 studies [25,27,28,31-33,35] did not specify the communication channels used by users to interact with the robots.

#### Functions and Roles of SARS

Content analysis identified and categorized 4 functions of SARs in physiotherapy interventions and activities: coaching, demonstration, peer support, and monitoring. However, this was based on data from the 12 included studies [4,24-31,33-35], as 1 study did not explicitly report the function of the SARs [32].

Coaching was identified as a prominent function of SARs in physiotherapy interventions, emphasizing their capacity to provide instructions and feedback. For example, in the study by Buitrago et al [24], the coaching role of SARs was as “[robot] gives feedback to the child by saying positive sentences for having completed the task’ and ‘[robot] verbally indicated him [user] extending an arm that he had to go to where the therapist was to bring another toy.” In some studies, the robots delivered game instruction and feedback to users using both verbal and visual feedback, encouraging users to improve their performance [29]. In other studies, the robot encouraged users during sessions using motivational phrases such as “Let’s go! You can do it!” [26].

Demonstration was identified as the second most common function reported (6/13, 46%), highlighting the role of SARs in physically showing or modeling exercises, techniques, and movements. This function was often described as leveraging the robot’s humanoid embodiment to demonstrate gestures and motions. In 5 studies [4,27,31,33,34], SARs like NAO or Poppy demonstrated exercises or performed them alongside patients. In a study by Cespedes et al [25], the robot provided posture feedback to a patient during gait training and demonstrated corrective cervical or thoracic posture techniques.

Monitoring, 2 studies [25,26] highlighted the role of SARs in monitoring and assessing safety warnings and reporting emergencies to human therapists. In the study by Irfan et al [26], NAO could request the study participant report their level of physical exertion using the Borg scale [40], a numerical scale that measures perceived exertion during physical activity, and issue warnings about a high heart rate during treadmill exercises for patients with a history of myocardial infarction. Similarly, Cespedes et al [25] reported that when a heart rate alert was detected, NAO verbally confirmed the patient’s condition and alerted the therapist promptly.

The role of peer support provided by SARs involves the robot engaging with the patient as a supportive companion during therapy sessions, often providing positive reinforcement and encouragement. Most studies described this role through various forms of motivational feedback, such as verbal motivation or encouragement and positive feedback [25,31]. Examples of supportive messages included phrases like “I’m so happy to see you again” and “It is so nice that you came back” [30]. Irfan et al [26] also personalized feedback by including the patient’s name in supportive statements aiming to enhance the robot’s perceived sociability. In addition, Carrillo et al [4] reported that NAO incorporated humor and preprogrammed dialogue to build rapport with patients.

#### Key Stakeholder Involvement in SAR Design and Development

Out of the 13 studies [4,24-35] reviewed, 7 (54%) studies [4,26,27,29,31,33,34] reported involving physiotherapists or other health care professionals in the design of SARs or intervention protocols. Physiotherapists specifically contributed to designing exercise programs [27,31,34], patient selection [34], and robot vocal feedback messages [33]. Feingold-Polak and Levy-Tzedek [29] reported system improvements based on recommendations from a focus group of expert clinicians who work with stroke survivors, the intended target users of the SARs. Carrillo et al [4] highlighted the active engagement of therapists in both the design and development phases of the robot, allowing them to critique and explore the system’s physical and clinical capabilities and limitations.

Only 2 studies [4,35] involved clinical populations in the design of SARs or intervention protocols. Back et al [35] mentioned the continuous development of an exercise program based on user feedback. Carrillo et al [4] described several early robot-patient interactions during the design and development to obtain structured feedback for protocol refinement. However, most included studies either did not report clinical population involvement [24,25,27-33](9/13, 69%) or did not include clinical populations in the design process [26,34](2/13, 15%).

#### Involvement of Technical Support and Physiotherapist During the Intervention

Three studies [4,30,35] reported providing technical support during interventions. In the study by Back et al [35], a therapist was present to ensure the robot functioned properly. In Feingold-Polak et al’s study [30], a clinician was present during the intervention to address technical problems, such as replacing a malfunctioning tag reader. Carrillo et al’s study [4] required engineer intervention to restart the system due to battery drainage. While 1 study [27] explicitly reported no technical support, 9 studies [24-26,28,29,31-34] did not provide any information regarding technical support required during the SAR intervention.

Eleven studies [4,24-31,33,35] identified various roles of physiotherapists and medical staff during physiotherapy sessions. In some instances, physiotherapists directly participated in therapy by coordinating activities with the robots [24,25] or providing physical support to patients with neurological impairments [31]. Five studies [4,28,30,33,35] explicitly mentioned physiotherapists ensuring the proper operation of SARs [30,35], setting parameters [28,30,33], or arranging the environment to facilitate smooth sessions [4,30]. In the study by Pulido et al [27], the therapist configured personalized exercises based on patient profiles and monitored sessions via a graphical interface. Similarly, Salas-Monedero et al [28] described monitoring through sensors to facilitate timely interventions. In the study by Irfan et al [26], medical staff present during cardiovascular rehabilitation sessions provided immediate assistance during emergencies, monitored vital signs, and interacted with the robot. In another study, physiotherapists played an educational role by introducing patients to robots and explaining system capabilities and interactions before sessions [33].

### Methods of Evaluation and Outcome

Table 4 summarizes the evaluation methods for treatment effectiveness reported in the included studies [4,24-35]. The outcomes are categorized into 2 groups. The first group includes clinical outcomes, user performance, or functional measures, which assess the direct impact of interventions on the user’s health and functional abilities, as well as their ability to perform tasks related to their functional goals. For example, Buitrago et al [24] used Goal Attainment Scaling [41] to evaluate the extent to which the patient with CP achieved his goals, with positive results indicating that the user successfully met the objectives during robotic training. Salas-Monedero et al [28] assessed upper extremity dexterity in patients with tetraplegia and paraplegia using metrics like the Box and Block Test [42]. Irfan et al [26] monitored physiological parameters, such as heart rate, the Borg scale [40], and blood pressure in a patient with a history of heart attack to observe the intervention’s effects.

The second group encompasses metrics that evaluate the acceptance, usability, and perception of robots. Among these, acceptance was the most frequently assessed construct, typically measured through subjective evaluations, such as interviews and questionnaires, with reports indicating a positive trend toward acceptance of SARs [4,26,29-31,33]. In addition, studies focusing on user perception reported positive feedback regarding the overall robot experience [35], usability [27], and increased user motivation and adherence to therapy [4,26,27,32]. However, 1 study found no improvement in adherence [33].

Although most of the included studies assessed the acceptance, usability, and perception of robots, 7 studies [4,29-32,34,35] did not report any clinical outcomes, user performance, or functional measures. This may limit the understanding of the actual therapeutic benefits of the robotic interventions.

**Table 4.** Methods of evaluation and key findings from included studies.

Author and year	Measures (clinical outcomes, user performance, or functional measures)	Measures (acceptance, usability, or perception of robots)	Key findings
Meyns et al [32], 2019	Not applicable	Motivation: Fun Toolkit (the Again table, the Smileyometer, and the Fun Sorter)	Humanoid robot instructors, like using music, enhance children’s initial motivation for physical activity programs.
Bäck et al [35], 2013	Not applicable	Feedback on robot experience: Survey questions	Positive feedback supports using NAO robots as exercise trainers and in nursing home rehabilitation for older adults.
Al‑Shahry and Al‑Shehri [34], 2020	Not applicable	Robot-patient performance: Clarity, therapy sequences, interaction, voice, timing, independence, operation and technical performance, and degree of freedom	Robot therapy shows highly satisfactory performance with good patient acceptance.
Butchart et al [31], 2021	Not applicable	Perceptions of acceptability and therapeutic value: Semistructured in-depth interview	NAO robot is an acceptable complement to rehabilitation therapies. NAO robots have perceived therapeutic value through their potential to enhance engagement and rehabilitation program delivery, particularly in the absence of a physiotherapist.
Feingold-Polak et al [30], 2021	Not applicable	User acceptance of the novel system: User satisfaction evaluation questionnaire and open-ended questionnaire Evaluation of technical limitations: Custom-made questionnaire	Long-term stroke rehabilitation with robotic platforms in clinical settings is feasible and shows strong acceptance among patients with poststroke.
Feingold Polak and Tzedek [29], 2020	Not applicable	Acceptance: Robot Acceptance Survey (RAS), a custom-made user-acceptance questionnaire based on the User Satisfaction Evaluation Questionnaire (USEQ)	Patients with poststroke demonstrate acceptance trends toward SAR after long-term interaction.
Martí Carrillo et al [4], 2018	Not applicable	Acceptance: Robot acceptance questionnaire from physiotherapists, acceptance questionnaire for guardians	Despite its limited capabilities, NAO’s robust minimalist system deployment provides valuable patient engagement insights for ongoing clinical deployment, especially for a formal clinical evaluation.
Buitrago et al [24], 2020	Effectiveness of intervention: Objective evaluation of intervention using the Goal Attainment Scale (GAS)	Not applicable	Participant showed increased continuous steps during training with a humanoid robot.
Salas-Monedero et al [28], 2023	User performance: Length of the hand trajectory (mm), peak number detected in the velocity profile of hand during the execution of the task (units), Box and Block Test (blocks)	Compliance: Record the number of sessions for each participant	Intervention benefited patients with tetraplegia by improving hand motor accuracy and dexterity. However, patients with paraplegia showed no improvement with intensive short-term training.
Irfan et al [26], 2020	Physiological parameters: Heart rate, Borg scale, gait parameters (step length, cadence, and speed), cervical posture, systolic blood pressure	Social interaction: Gaze and patient’s verbal and nonverbal feedback Technology use and acceptance: Unified Theory of Acceptance and Use of Technology (UTAUT) questionnaire	Improved physiological progress observed. Patient adherence to the cardiac rehabilitation program may have been enhanced with personalized robot attendance.
Cespedes et al [25], 2020	Patient performance: Heart rate, Borg scale Poor posture time (cervical and thoracic): The total time during the session when the posture inclination overcomes the good posture threshold	User perception: Questionnaire to the patient and the therapist	SAR^a^ had a positive effect on patient encouragement, intrinsic motivation, and adherence to the therapy and was well-received by patients and therapists, with reported benefits in gait rehabilitation sessions.
Pulido et al [27], 2019	Motor performance: Manual Ability Classification System (MACS), Motor scale of Mallet, Quality of Upper Extremity Skills Test (QUEST) scale	Usability and compliance: Record the number of sessions for each participant User perception on satisfaction and usability: Questionnaires for patients, relatives, and clinical staff, Likert scale (5 items), open questions	Patients showed slight motor skill improvement with robotic platform training compared to conventional therapy (where no improvements were detected) Deemed useful and easy to operate by patients, relatives, and health care professionals.
Blanchard et al [33], 2022	Change in lumbar pain: Visual analog scale (VAS) Change in disability: Roland Morris Questionnaires (RMQ), the Dallas Pain (DPQ) Questionnaires Adverse events: Reported by physiotherapists during therapy sessions and by investigators during evaluation visits	Adherence: Record number of movements performed by the participants during each robot-led session Change in fears and beliefs: Fear-Avoidance Beliefs Questionnaire (FABQ) Usability: System Usability Scale (SUS) Therapist’s acceptance of robot: Unified Theory of Acceptance and Use of Technology (UTAUT) questionnaire	Adherence to robot sessions declined over time, limiting physical activity supervision. No negative impact is shown on clinical outcomes, but the system was deemed not yet ready for rehabilitation applications.

a SAR: socially assistive robotic.

### Reported Limitations and Areas of Improvement

Overall, 4/13 (31%) included studies [28,29,31,35] reported a limitation related to small sample sizes, thus limiting the generalizability of their findings. Selection bias was identified as a potential limitation in 1 study [31], where researchers may have been inclined to select participants they had previous positive interactions with. Other study limitations identified included slow recruitment rates due to strict inclusion criteria [33] and the homogeneity of participants from the same facility [29]. Technical issues and limitations in robot design were reported in 3/13 (23%) studies [33-35], such as system readiness issues and malfunctions leading to missed robot sessions [33], pelvic movement readiness and system heating concerns [34], and limited exercise variety due to robot size constraints [35].

In addition, 3/13 (23%) studies [4,24,34] highlighted limitations in intervention design. For instance, Buitrago et al [24] noted that, although the objective measured by the Goal Attainment Scaling was achieved during the training, neither the robot nor the therapist adjusted the task or the environment to reflect the new goal. Carrillo et al [4] reported that the therapist performed tasks for the robot as they were not informed of the robot’s capabilities. Other reported limitations included a limited range of friendly interactions, the need for refreshment breaks, and limited flexibility in exercise sequences [34].

Other identified limitations included robotic technology expenses [35] and uncertainty regarding the impact of color blindness in users [30]. Identified limitations in evaluation methodologies included difficulty in measuring motivation in very young children [32], a lack of assessment of the robot’s impact on physical health [33,35], and the absence of cognitive ability data to indicate acceptability [31]. Three studies [25-27] did not report any limitations.

The areas of application proposed by the authors of the included studies spanned the domains of rehabilitation, such as cardiac rehabilitation [26], pediatric oncology, and neurorehabilitation [24,28,30,32], and applications in older adult care facilities [35]. Other identified applications included supporting exercise, progress monitoring, and maintaining motivation and adherence [26]. In addition, studies’ authors suggested integrating SARs into existing robot-assisted gait rehabilitation [25], using SARs to address gaps in telehealth care by enabling real-time program adjustment based on robot-patient interactions [34], and supporting long-term home exercise programs for people with chronic conditions who may not have access or resources for prolonged therapist contact to assist with managing their condition [33]. One study [29] did not propose any specific areas of application.

## Discussion

### Main Findings

This scoping review aimed to explore the usage of SARs in physiotherapy contexts with clinical populations. Across the 13 studies [4,24-35] identified, SARs’ roles and functions were tailored to suit the specific needs of patients and the objectives of physiotherapy interventions. These findings indicate that, in current applications, the primary objective of integrating SARs into health care is therapeutic augmentation [43].

Numerous studies reported that the use of SARs led to repetitive exercises and sessions [44]. This issue may stem from simplistic programming or a lack of personalized adaptation, which can result in a loss of user interest [44]. To address this, efforts have increasingly focused on complementing SAR interventions with devices like Lokomat or incorporating more advanced gamified exercises [4,25,27,29,30]. This review identified several SAR capabilities, including coaching, demonstration, monitoring, and peer support. Currently, SARs seem to be prioritized for roles, such as time-extending coaching and monitoring, functions that are considered unfeasible for human therapists due to the mismatch between the number of available therapists and the size of clinical populations [43].

### Robots Used

The dynamics of socially assistive interaction are elucidated through the roles that SARs take on and the communication channels used between robots and users. In all but 2 studies [28,32], where communication methods were not reported, verbal communication channels were used to deliver feedback, encouragement, or instructions (along with visual and motion-related communication). Verbal praise to users is considered positive feedback and thus has the potential to enhance users’ intrinsic motivation for task performance [43].

Of the SAR models discussed in the review, the humanoid robot NAO is the most frequently used. Although there is limited evidence guiding the selection of the most suitable SAR model for physiotherapy applications, NAO has been commonly adopted in aged care and pediatric rehabilitation contexts, noted for its good acceptance by users and physiotherapists and movement capabilities [13,45]. The prevalent use of NAO in clinical settings may stem from its perceived usefulness in prior feasibility tests [46], its commercial availability and its well-established functional and technical capabilities [47].

### Stakeholder Involvement in the Design of SARs and Intervention Protocols

The involvement of domain experts (physiotherapists) and end users (clinical populations) in the design of the SAR system and the intervention protocols was investigated, with a focus on understanding whether the systems adequately meet the needs of important stakeholders. The findings show a lack of participation from key stakeholders in both processes. Among the 13 included studies [4,24-35], only 2 studies [4,35] appeared to have involved end users in the design and development phase, while the remaining studies either did not report or did not engage stakeholders during these stages. In Back et al’s study [35], participants provided feedback on exercise programs to improve their effectiveness. In Carrillo et al’s study [4], the authors emphasized the early engagement of patients and physiotherapists in the design and development of SARs, highlighting the benefits of overcoming technological limitations, fostering effective engagement with end users, and facilitating the development of the robot’s demonstration function. A previous study has identified the value of stakeholder input in robot development, as it aids in identifying potential uses for developers, pinpointing flaws in current robot designs to avoid, and suggesting improvements to ensure usability, particularly in health care settings [48].

### Limitations in the Current Deployment of SARs With Clinical Population

The majority of studies reported limitations within their findings, particularly regarding small sample size [28,29,31,35], challenges in patient recruitment [31,33], the robot’s design and technical capabilities [33-35], intervention protocol design [4,24], and evaluation methods [24,32,33]. Meanwhile, despite significant efforts in the application of SARs in physiotherapy, many of the studies are primarily pilot studies intended to pave the way to clinical trials with end users. However, they often fail to progress into larger studies due to the various limitations highlighted in Lo’s [49] paper, such as the inappropriate selection of control groups to assess therapeutic benefits, the absence of power calculations to detect minimally clinically important differences, and insufficient detail in robot rehabilitation protocols, hindering reproducibility. The current included studies have flaws, such as slow patient recruitment processes [31,33] and small sample size [28,29,35]. This could potentially compromise the validity of these trials and their impact on the clinical evaluation of the effect of SARs on patient outcomes.

Other limitations stem from the shortcomings of the current SAR designs and technological limitations, such as pelvic movement robustness, system overheating [34], robot size that affects exercise variety [35], and system readiness and malfunction [33]. This aligns with a study that investigated NAO-assisted pediatric CP rehabilitation, where most physiotherapists considered system or hardware failures during sessions as crucial features needing improvement [50].

In addition, the personalization of the robots to adapt to the user’s mood, fatigue, and performance variations during long-term involvement, such as in rehabilitation, remains a major challenge. In a review analyzing patients’ and clinicians’ opinions on the use of SARs in rehabilitation, the loss of user interest frequently arose as a concern [51]. This could pose a challenge in applying SARs when working with vulnerable user populations, supporting self-rehabilitation, or long-term exercise programs for chronic conditions [33].

Previous papers have described several barriers to the integration of SARs in clinical settings [44,49]. When recruiting end users (clinical populations) in clinical trials, several ethical issues must be carefully considered regarding patient physical and psychological safety, especially when involving vulnerable clinical populations (eg, children or people with cognitive impairments) [52]. Another significant hurdle is justifying the investment in clinical SAR research without clear economic or clinical evidence of its cost-effectiveness, which may impede the adoption of SARs in clinical settings. In addition, conducting research in the clinical setting can be time-consuming, as it requires multidisciplinary collaboration between robotics and health science experts who are rarely co-located [43]. These factors may indeed contribute to the high budget requirements of robot research in clinical settings.

### Strengths and Limitations of This Scoping Review

A strength of this scoping review is the interdisciplinary review team with expertise in the fields of physiotherapy (JH and MF), robotics (PCM), and scoping review methodologies (MF). This interdisciplinary perspective was important for identifying relevant locations to search, terms to use, and knowledge of disciplinary publication conventions. While numerous medical and engineering databases were comprehensively searched, citation tracking was limited to short reports that otherwise met the inclusion criteria for pragmatic reasons. This may have resulted in the omission of potential studies suitable for inclusion, but this risk is considered minimal given that the citation tracking found no further eligible studies for inclusion.

Furthermore, one team member led the majority of title and abstract screening, data extraction, synthesis, and analysis. This approach may introduce bias into the study exclusion, data extraction, and synthesis processes, potentially leading to incomplete data capture due to the risk of overlooking relevant information and nuances [53]. The primary reviewer’s limited experience in scoping review methodology may further compound these potential biases [53]. However, this risk was counterbalanced in this review through the auditing of processes and data, the use of collaborative review tools with clear audit trails, and regular meetings for confirmation of decisions and team endorsement to progress after each review stage.

### Conclusions

This scoping review identified what is currently known about the usage of SARs in physiotherapy interventions and activities with clinical populations. Despite the limited publications on this topic, the review offers insights into SAR design and application areas in physiotherapy interventions. This review has highlighted the future potential of SARs to be increasingly applied in health care settings. Future research should prioritize addressing the limitations in intervention design and evaluation methods to enhance the quality of evidence in this field. There is a critical need to resolve technical issues associated with SARs, such as system readiness, malfunction, size restriction, and overheating, to enhance the quality of interventions. Engaging key stakeholders, including physiotherapists and clinical populations, at all stages of SAR design and development is essential to overcoming technical challenges and promoting user acceptance. In addition, conducting more clinical trials and using reliable and valid clinical outcome measures are essential steps to investigate the effectiveness of SARs in promoting health outcomes.

## Supplementary material

10.2196/69908 Multimedia Appendix 1 Complete search terms for socially assistive robotics and physiotherapy.

10.2196/69908 Multimedia Appendix 2 Overview of used search strings and number of publication 'hits' (Results).

10.2196/69908 Checklist 1 PRISMA Checklist.

## Abbreviations

ACM: Association of Computing Machinery
CP: cerebral palsy
ICF: International Classification of Functioning, Disabilities and Health
IEEE: Institute of Electrical and Electronics Engineers
MeSH: Medical Subject Headings
PCC: population, concepts, context
PRISMA-ScR: Preferred Reporting Items for Systematic Reviews and Meta-analyses for Scoping Review
SARs: Socially Assistive Robots
WHO: World Health Organization
